# Maternal and Neonatal Outcomes of COVID-19 in Pregnancy: A Single-Centre Observational Study

**DOI:** 10.7759/cureus.13184

**Published:** 2021-02-06

**Authors:** Vinita Singh, Anisha Choudhary, Mamta R Datta, Alokananda Ray

**Affiliations:** 1 Obstetrics and Gynecology, Tata Main Hospital, Jamshedpur, IND

**Keywords:** sars-cov-2, pregnancy, maternal outcome, neonatal outcome, vertical transmission, covid 19

## Abstract

Background: The current coronavirus disease 2019 (COVID-19) pandemic is one of the most challenging healthcare crises faced globally. Adequate information and understanding of the clinical presentation and impact of the disease on maternal and neonatal outcomes is the key to successfully manage a pregnancy with COVID-19.

Objective: The purpose of the present study was to evaluate the clinical presentation of COVID-19 in pregnancy, its course during pregnancy and its effects on maternal and neonatal outcomes.

Study design and setting: This study was a retrospective observational study conducted at Tata Main Hospital, Jamshedpur, a tertiary care hospital in Eastern India.

Population and study period: All COVID-19-positive (by reverse transcription polymerase chain reaction or rapid antigen test) pregnant women admitted to the hospital from 15^th ^May 2020 to 15^th^ November 2020.

Results: A total of 132 COVID-19-positive pregnant women were included in the study. Eighty-six women (65.15%) were asymptomatic, 45 women (34.09%) had mild symptoms and one woman had severe disease. Major co-morbidities seen were hypertensive disorders (pre-eclampsia, gestational hypertension and chronic hypertension) in 18 (13.64%) and diabetes (gestational diabetes, diabetes mellitus type 2) in 14women (10.60%). The rate of preterm delivery was 28.69% (n=35). Caesarean section was done for 78 women (63.93%) and 44 (36.07%) delivered vaginally. Average birth weight reported was 2.59 kilograms. Forty babies (33.06%) were admitted to the neonatal intensive care unit. Two babies (1.65%) tested positive for severe acute respiratory syndrome coronavirus 2 (SARS-CoV-2) within 24 hours of delivery.

Conclusion: COVID-19 in pregnancy commonly presents as an asymptomatic or mild disease. It is associated with high rates of preterm births and neonatal admissions to the intensive care unit. Intrauterine and neonatal death rates remain low. Vertical transmission is possible; however, the incidence is low, and the majority of these neonates are asymptomatic.

## Introduction

The ongoing coronavirus disease 2019 (COVID-19) pandemic caused by severe acute respiratory syndrome coronavirus 2 (SARS-CoV-2) has resulted in a global healthcare crisis.

Owing to its novelty, limited knowledge is available about the effect of COVID-19 disease on pregnancy outcome. The knowledge obtained from previous human coronavirus outbreaks like Middle East respiratory syndrome (MERS) and severe acute respiratory syndrome coronavirus (SARS-CoV) tends to lean towards poorer outcome in pregnant women when compared to the general population [1,2]. Earlier reports [3,4] did not show any increased adverse effect of COVID-19 on pregnant women, however there is emerging evidence that shows higher risk of severe disease and increased Intensive Care Unit (ICU) admissions in pregnant women when compared to non-pregnant women [5,6].

This study aims to add to the existing knowledge about the maternal and perinatal outcomes of pregnant women infected with SARS-CoV-2.

## Materials and methods

This is a retrospective analysis of prospectively collected data of patients admitted from 15th May to 15th November 2020 in the Department of Obstetrics and Gynecology, at Tata Main Hospital, a tertiary care hospital in Jamshedpur, Jharkhand, India. It is a designated COVID-19 care hospital running the largest COVID-19 labor room and emergency services in the city.

All pregnant women admitted in the hospital were tested according to Indian Council of Medical Research (ICMR) guidelines which states that pregnant women residing in clusters/containment areas or in large migration gatherings/evacuee centers in hotspot districts and presenting in labor or likely to deliver in five days should be tested for COVID-19, even if asymptomatic. The samples were collected either on out-patient basis or after admission, in accordance to Ministry of Health and Family Welfare, Government of India guidelines [7].

All women were first seen in a triage area and transferred to negative, suspect or positive isolation wards according to their COVID-19 screening status. All health care workers (HCWs) wore the appropriate personal protective equipment (PPE) according to their area of duty and followed social distancing protocols.

Positive women were managed in consultation with a physician and transferred to a positive isolation ward or ICU as per disease severity. Asymptomatic women and those with mild symptoms were discharged after 24 hours of vaginal delivery and 48 hours of caesarean section with advice of home isolation. All neonates were tested within 24 hours of delivery. Rooming in and breastfeeding were allowed, and all mothers were encouraged to follow hand hygiene, wear masks and follow social distancing protocols.

Data of 132 COVID-19-positive (reverse transcription polymerase chain reaction [RT-PCR] or rapid antigen test) pregnant women admitted in the hospital within the study period of six months (15th May 2020 - 15th November 2020) was retrospectively entered in a Microsoft Excel sheet. Details about their age, period of gestation, history of contact, symptoms, associated comorbidities, mode of delivery and baby details were collected. Continuous variables are expressed as mean +/- Standard Deviation (SD). All categorical variables are expressed as frequency and percentages.

## Results

A total of 2,729 pregnant women were admitted in the Obstetrics and Gynecology Department from 15th May to 15th November 2020. Out of these, 132 women (4.83% incidence) were confirmed COVID-19-positive by molecular detection of SARS-CoV-2 by RT-PCR or rapid antigen test. A total of 122 women delivered, six women had ongoing pregnancy, three had spontaneous early pregnancy loss, one had laparotomy in view of ruptured ectopic pregnancy. Out of 132 COVID-19-positive women only 12 gave history of contact, and two had history of recent out-of-state travel. In the rest of the 118 women (89.39%) source of infection could not be identified and was possibly via a community transmission.

Study characteristics

**Figure 1:**
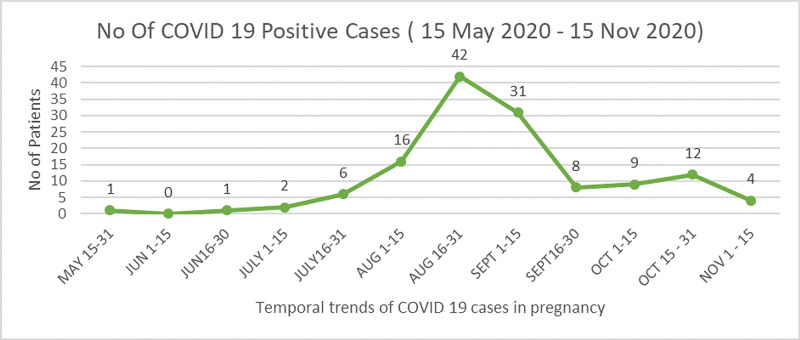
Temporal trends of coronavirus disease 2019 (COVID-19) cases in pregnant women during our study. NOV - November, JUN - June, AUG - August, SEPT - September, OCT - October

The first case of a confirmed COVID-19-positive patient was admitted on 21st May 2020. There was an exponential rise in the number of cases during the month of August, reaching a peak during 15th August to 31st August, followed by a decline. A small rise was again observed during the month of October which is the festival season in the Indian subcontinent followed by a decline, as depicted in Figure 1.

The demographic profile of the patients is depicted in Table 1. The patient’s age ranged from 17 years to 41 years with maximum number of women in the age group of 21-30 years. Mean age was 27.5+/- 4.57 years. The number of primigravida were 65 and 67 were multigravida. Majority of the women presented in the third trimester.

**Table 1:** Demographic Profile of the Patients.

Parameters	Number of patients (n)	Percentage (%)
Age Group (years)		
< 20	8	06.06
21–30	97	73.48
31–40	26	19.70
> 40	1	00.76
Total	132	100
Parity		
Primigravida	65	49.24
Multigravida	67	50.76
Total	132	100
Period of gestation (in weeks)		
≤12 weeks	5	03.79
12 – 28 weeks	2	01.51
>28 weeks	125	94.70
Total	132	100

Maternal presenting symptoms and associated co-morbidities

Approximately 65.15% of women (n=86) were asymptomatic and 45 (34.09%) women had mild symptoms of cough, fever, rhinitis, myalgia or fatigue. One primigravida was admitted at 29 weeks of gestation with COVID-19 pneumonia and features of possible viral meningo-encephalitis. She was admitted in COVID-19-positive ICU and required non-invasive ventilation support for 10 days, she was also given injection dexamethasone, low molecular weight heparin (LMWH) and injection remdesivir. She tested COVID-19-negative on day 14 and was discharged on that day in a stable condition and an ongoing pregnancy. Detailed breakdown of maternal co-morbidities is given in Table 2. Major co-morbidities seen were hypertensive disorders (pre-eclampsia, gestational hypertension and chronic hypertension) in 18 women and diabetes in 14 (gestational diabetes, diabetes mellitus type 2). Approximately 24% of our patients had deranged liver enzymes. Thirteen women had anemia and 11 of them needed blood transfusion. One woman was morbidly obese and was given prophylactic LMWH.

**Table 2:** Maternal Symptoms and Co-Morbidities. HTN – Hypertension, GHTN – Gestational Hypertension, DM – Diabetes Mellitus, GDM – Gestational Diabetes Mellitus.

Parameters	Number of patients (n)	Percentage (%)
Symptoms at admission		
Asymptomatic	86	65.15
Mild	45	34.09
Severe	1	00.76
Total	132	100
Maternal Co-morbidities		
Deranged liver enzymes	32	24.24
HTN/Pre-eclampsia/GHTN	18	13.64
DM/GDM	14	10.60
Anemia	13	09.85
Thrombocytopenia	10	07.57
Hypothyroidism	08	06.06
Morbid Obesity	01	00.76

Pregnancy outcomes

Out of the total 132 pregnant women who tested SARS-CoV-2-positive during the study period, 122 delivered, six had ongoing pregnancy and were managed conservatively, three women had spontaneous first-trimester miscarriage (two underwent suction evacuation whereas one had complete miscarriage), and one had laparotomy in view of ruptured ectopic pregnancy. We also saw a high rate of preterm deliveries, with 35 patients (28.69%) delivering before 37 weeks. The preterm delivery rate during the same time period in the preceding year was 13.28%. As depicted in Table 3, 63.93% (n=78) women had caesarean section and 36.07% (n=44) delivered vaginally. The most common indication of lower segment caesarean section (LSCS) was previous caesarean section followed by fetal distress and failed induction of labor. Five women (6.75%) refused trial of labor and wanted caesarean sections.

**Table 3:** Pregnancy Outcome IUGR - Intra Uterine Growth Retardation

Parameters	Number of patients (n)	Percentage (%)
Time of Delivery		
Preterm Birth (< 34 weeks)	7	05.74
Preterm Birth (34 – 37 weeks)	28	22.95
> 37 weeks	87	71.31
Total	122	100
Mode of Delivery		
Vaginal delivery	40	32.79
Forceps assisted delivery	4	03.28
Lower Section Caesarean Section	78	63.93
Total	122	100
Indication for Lower Section Caesarean Section		
Previous Caesarean Section	25	32.05
Foetal distress	20	25.64
Failed Induction of labour	13	16.67
Primigravida with breech presentation	6	07.69
Not willing for trial of labour	5	06.41
Twins with 1^st^ non-cephalic presentation	02	02.56
Mono Chorionic Di Amniotic twin	01	01.28
Cephalopelvic disproportion	02	02.56
Non-progress of labour	01	01.28
IUGR with severe oligohydramnios	03	03.85
Total	78	100

Maternal complications

Table 4 shows various maternal complications seen in our patients. Seven women had postpartum hemorrhage and one patient had antepartum hemorrhage requiring blood transfusion. One woman had vulval hematoma post vaginal delivery, which was repaired in Operating Room. One patient had severe preeclampsia and was managed with magnesium sulphate infusion. Severe maternal complication was seen in two women. First was a primigravida at 29 weeks gestation admitted with acute breathlessness and seizures, pneumonia and features suggestive of possible viral meningo-encephalitis. Second was a primigravida who developed sepsis with multiorgan involvement and COVID-19 pneumonia post LSCS for twin pregnancy with both fetuses in breech presentation. Both patients were managed in intensive care unit on non-invasive ventilation, injection remdesivir, injection dexamethasone and LMWH. Both were discharged in a stable condition. There was no case of maternal mortality during our study period.

**Table 4:** Maternal Complications MODS – Multi Organ Dysfunction Syndrome

Parameters	Number of patients (n)	Percentage (%)
Maternal Complications		
Post-partum haemorrhage	7	05.30
Ante-partum haemorrhage	1	00.76
Sepsis with MODS	1	00.76
COVID 19 pneumonia with seizures	1	00.76
Vulval haematoma	1	00.76
Severe Preeclampsia	1	00.76
No complications	120	90.91
Total	132	100

Neonatal outcomes and complication

Among the 122 pregnant women who delivered, three had twin babies, making the total births 125 babies. Four women were diagnosed with intrauterine death on admission, and none of them were registered for antenatal care in our hospital from before. One hundred twenty-one live births were reported. In terms of neonatal outcomes, the majority of the babies did well as depicted in Table 5. Birth weight of the babies ranged from 0.8 to 4.25 kilograms (kg) with an average birth weight of 2.59 kg. Forty (33.06%) babies were admitted to the neonatal intensive care unit (NICU). Seven babies were intubated, three of which died, three were discharged and one was transferred to another center. All three neonatal deaths were of extremely low birth weight babies (<1.5 kg) with severe sepsis. Seventy-eight babies were roomed in with the mother, and 36 babies from NICU were discharged. Nasal swabs were performed on all 121 babies within 24 hours of delivery to screen for SARS-CoV-2. Two babies (1.65%) tested positive, they were asymptomatic and roomed in with their mothers. One baby was delivered vaginally and one via LSCS. Both their mothers had mild symptoms.

**Table 5:** Neonatal Outcome and Complications NICU – Neonatal Intensive Care Unit

Parameters	Number of babies (n)	Percentage (%)
Neonatal Outcome		
Intra Uterine Foetal deaths	04	03.20
Live births	121	96.80
Total births	125	100
Birth Weight (Kilograms)		
<1	02	01.65
1 – 1.4	01	00.83
1.5 – 1.9	08	06.61
2 – 2.4	24	19.83
2.5 – 2.9	47	38.84
3 – 3.4	26	21.49
3.5 – 3.9	12	09.92
≥ 4	01	00.83
Total	121	100
Neonatal Complication		
NICU admissions	40	33.06
Neonatal Deaths	03	02.48
COVID Positive	02	01.65
Total Live births	121	100

## Discussion

The novel coronavirus infection has put an unprecedented and unimaginable dent in our healthcare systems. Pregnant women are considered a vulnerable and high-risk group for COVID-19 infection due to the fear of its effect on pregnancy and the new-born. The purpose of this study was to understand the clinical spectrum, maternal and neonatal impact of COVID-19 on pregnant women.

In our study, 132 COVID-19-positive mothers were included, the majority (94.69%) of whom were in the third trimester. Eighty-six patients (65.15%) were asymptomatic, 34.09% had a mild form of the disease and only one presented with severe disease requiring intensive care. Similar findings were seen in a study of 141 patients in Mumbai where most of their patients (97%) were asymptomatic or had mild symptoms [8]. A living systematic review and meta-analysis in British Medical Journal also stated that symptoms of fever and myalgia manifest less often in COVID-19-infected pregnant women than in nonpregnant women [5]. A higher percentage of asymptomatic presentation can also be due to the strategy of universal testing for COVID-19 in pregnancy and lower thresholds of testing than in the general population. As this testing often occurs near term, most of these women are in the third trimester and asymptomatic positive women in early pregnancy are likely to be missed.

Fortunately, we had only two patients who had severe presentations requiring intensive care and no case of maternal mortality was seen among our patients. Similarly, few studies have [3,4,8,9] have also reported that COVID-19 does not have more severe presentation in pregnancy. However, emerging studies have shown higher risk of severe disease and increased ICU admissions in pregnant women when compared to non-pregnant women [5,6,10].

In our study, hypertensive disorders (13.28%), diabetic disorders (10.15%), and anemia (10.15%) were the most commonly seen co-morbidities. Similarly, Nayak et al. [8] and Gajbhijye et al. [11] also reported hypertensive and diabetic disorders as the most commonly associated co-morbidities. In a prospective cohort study by Antoun et al., 10.5% of patients had severe pre-eclampsia compared to 1-2% risk in the general population, out of which one patient developed hemolysis, elevated liver enzymes and low platelet count (HELLP) syndrome and disseminated intravascular coagulation [12]. One possible explanation can be that placental angiotensin-converting enzyme 2 (ACE2) is highly expressed at the maternal-fetal interface and its dysregulation by SARS-CoV-2 might be involved in the high rates of preeclampsia associated with severe and critical COVID-19-infected pregnant women [13]. A quarter of our patients had abnormal liver functions. As serum bile acid levels were not measured for these patients it is difficult to comment whether the hepatic dysfunction was solely due to COVID-19 or had an overlap with obstetrics cholestasis. Few studies have reported abnormal liver function tests in a high number of their study subjects [14,15], however all these studies were conducted among non-pregnant COVID-19 infected adults and whether the incidence of hepatic dysfunction is higher among COVID-19 infected pregnant females is yet to be determined.

In our study, out of 132 COVID-19-positive women, 122 delivered, six had ongoing pregnancy, three had spontaneous first-trimester miscarriage and one had laparotomy in view of ruptured ectopic pregnancy. LSCS was done for 78 women, making our caesarean section rate 63.93% (78/122). Most of the earlier studies on COVID-19-positive pregnant women report very high rates of caesarean sections, where the majority of LSCS were done in maternal interest, due to concern for respiratory function [16-19]. COVID-19-positive as a sole indication for LSCS has come down in recent studies. Nayak et al. reported a 50% LSCS rate in COVID-19-positive women which was not significantly higher than the COVID-19-negative group [8].

In our study too all the caesarean sections were done for obstetric indications only, maximum being for previous caesarean sections in labor (32.05%). Due to limitations in the COVID-19-positive isolation ward, we did not give trial of labor to pregnant women with previous caesarean section. Other main indications were fetal distress (25.64%) and failed induction of labor (16.67%). A plausible explanation for the high number of failed inductions may be that these patients were in isolation, away from family, and were being looked after by HCWs in PPEs who failed to develop patient-HCW bonding, resulting in several women opting out of induction process halfway.

In our study, we encountered a high rate (28.69%) of preterm births (35/122). Spontaneous preterm births were 21.31% (26/122), six were terminated due to fetal distress, two due to severe intra uterine growth retardation (IUGR) and one because of severe preeclampsia. In our study, preterm premature rupture of membranes (PPROM) was seen in 8.4% of patients. Many reviews report high rates of preterm deliveries among COVID-19-affected pregnant women ranging from 41% to 47% [2,18] but the cause for the high preterm births remains unclear in these studies. A living systematic review by Allotey et al. has also concluded that pregnant women infected with COVID-19 are more likely to give preterm birth and have higher incidence of neonatal admissions to the intensive care unit [5]. However, a systematic review of 33 studies that described the outcomes of 385 pregnant women with COVID-19 had lower preterm birth rate of 15.2% and most of these deliveries were iatrogenic and for maternal interest [19]. At present, there is insufficient evidence to determine any correlation between spontaneous preterm labor and COVID-19 infection in pregnancy.

Along with high number of preterm births we also saw high incidence (29%) of low birth weight babies and NICU admissions (33.33%). However, incidence of intrauterine deaths and neonatal deaths remained low with 3.20% and 2.48% respectively. In corroboration with our findings, Nayak et al. also reported low birth weight in 29.77% and intrauterine death in 2.23% of COVID-19-positive pregnant women included in their study [8]. Allotey et al. also concluded that although pregnant women with COVID-19 are more likely to experience preterm birth, low birth weight babies and higher number of neonatal admissions, the overall rates of stillbirths and neonatal deaths is not higher than the background rates [5].

Earlier research had suggested that the SARS-CoV-2 infection is not transmitted from the mother to child and is not detected in placenta, amniotic fluid, cord blood, and neonatal throat swab samples [17,20]. However, there is emerging evidence that now suggests that vertical transmission is possible. Two reports have published evidence of immunoglobulin M (IgM) for SARS-CoV-2 in neonatal serum at birth [21,22]. Facchetti et al. reported that maternal-fetal transmission of SARS-CoV-2 is likely to be propagated by circulating virus-infected fetal mononuclear cells [23]. Hosieret et al. confirmed SARS-CoV-2 invasion of the placenta, predominantly localized to syncytiotrophoblast cells at the maternal-fetal interface of the placenta [24]. In a large systematic review of 666 neonates born to women with confirmed COVID-19, 28/666 (4.2%) neonates had confirmed COVID-19 infection postnatally [25]. In our study, nasal swabs were taken from all 117 babies within 24 hours of delivery to screen for SARS-CoV-2 and two babies (1.70%) tested positive (one delivered vaginally, one via caesarean section). Both babies were asymptomatic and roomed in with the mother and were discharged with the mother.

## Conclusions

In the present study, we did not find COVID-19 in pregnancy to have a severe clinical presentation. There is a high rate of preterm delivery, low birth weight and neonatal admissions, but the incidence of intrauterine and neonatal death is low. Neonatal COVID-19 infection is possible but uncommon, mostly asymptomatic, and the rate of infection is no different whether the baby is born vaginally or by caesarean section. As most of our patients presented to us in the third trimester, whether COVID-19 has any teratogenic effects could not be ascertained. As the disease is new, long-term follow-up is required to study any residual or delayed effects on the new-born. Although our cohort of 132 women is small, it can provide useful information to enhance our existing knowledge about COVID-19 infection in pregnancy and help in development of antenatal counselling and management protocols to achieve a safe and favorable maternal and neonatal outcome.
